# Genotypes analysis and antifungal susceptibility of *Candida albicans* strains isolated from women with vaginal candidiasis in Jordan using PCR targeting 25SrDNA and ALT repeat sequences of the RPS

**DOI:** 10.12669/pjms.40.8.9811

**Published:** 2024-09

**Authors:** Rania M. Al-Groom, Rand Raid Mahmoud Ali, Qasem M. Abu Shaqra

**Affiliations:** 1 Rania M. Al-Groom, Department of Medical Laboratory Science, Faculty of Allied Medical Sciences, Zarqa University, Zarqa, Jordan; 2 Rand Raid Mahmoud Ali, Faculty of Graduate Studies, Al-Balqa Applied University, Salt, Jordan; 3 Qasem M. Abu Shaqra, Department of Allied Medical Sciences, Zarqa University College, Al-Balqa Applied University, Salt, Jordan

**Keywords:** Vaginal candidiasis, *Candida albicans*, genotyping, rDNA, RPS, Antifungal susceptibility

## Abstract

**Background & Objectives:**

Genotypic identification of the etiologic agents of vaginal candidiasis (VC) is of significance in epidemiologic studies and in the establishment of adequate treatment protocol. The aim of this study was to determine the antifungal susceptibility and gene diversity of *C. albicans* isolated from a group of Jordanian women with VC.

**Methods:**

A total of 312 isolates of candida species, recovered from women with vaginal candidiasis who attended gynecology clinics affiliated to three major private hospitals in Amman over a period of five months (July 2020 to December 2020) were included in this study. The isolated Candida were characterized by phenotypic and genotypic means. Genotypic studies were performed using specific PCR primers of the rDNA and RPS genes. Susceptibility testing of all *C. albicans* isolates was conducted following the National Committee for Clinical Laboratory Standards and E-test strips.

**Results:**

*Candida albicans* was the most dominant *Candida spp*. that caused VC among the studied population. *C. albicans* isolates were found to be of three different subtypes at the 25S rDNA gene. All isolates belonged to genotypes A, B and C while genotypes D and E were not detected. The diversity of *C. albicans* was higher on the basis of RPS region where the use of two markers (P-I and P-II) resulted in the identification of nine distinct *C. albicans* subtypes. The sensitivity testing revealed variations in the susceptibility of various genotypes to different antifungal drugs. Genotype A isolates were more susceptible to fluconazole, flucytosine and ketoconazole than genotypes B and C.

**Conclusion:**

*Candida albicans* incriminated as etiologic agents of vaginitis among Jordanian women exhibited relationship between various genotypes and antifungal drugs.

## INTRODUCTION

Vaginal Candidiasis (VC) is one of the most prevalent human fungal infections, and is estimated to affect approximately 75% of all women at least once in their lifetime.^1^,^2^ The infection is caused by a group of *Candida spp* with *C*. *albicans* being the most dominant, whereas, *Candida glabrata* is frequently reported as the second or third in prevalence.^3^ However, other *Candida spp*. can also cause the infection including *C. tropicalis, C. parapsilosis, C. krusei* and *C. dubliniensis*.^4^ The infection is characterized by a diversified symptom that includes; itching, edema, erythema of the vulva, white vaginal curdy discharge and pain.^5^

VC is rare before puberty; its first episode could be related with the onset of sexual activity and increases dramatically in the second decade of life and beyond. Although, Candida is a member of the normal flora in vagina and other sites of the human body, many predisposing factors can lead to infection such as pregnancy, long term use of antibiotics, diabetes, use of corticosteroids, HIV infection, and immunocompromised situations.^6^,^7^

Clinical symptoms alone cannot diagnose VC accurately. Other infections such as bacterial vaginosis and vaginal trichomoniasis can confuse the diagnosis of VC.^8^ Symptoms that may be prevalent in these two infections can be found in women with VC.^8^ Therefore, microbiological work up using culture methods is recommended for diagnosis.

The occurrence of VC in Jordan was the subject of few studies. These studies showed that *C. albicans* was by far the most dominant etiologic agent responsible for this infection. Study by Abu-Elteen et al.^9^, showed that *Candida albicans* was the dominant species which was isolated from 51.3% of patients, followed by *C. glabrata* “with an isolation rate of 17.9%”. However, the genetic makeup of these isolates was not determined.

The identification of *C. albicans* to the strain level is sometimes important because there is a correlation between the Candida genotype and antifungal susceptibility. Antifungal susceptibility test results indicated that isolates of genotype A of *C. albicans* were significantly less susceptible to flucytosine than either *C. albicans* genotype B or C.^10^-^12^ Another study from Nigeria showed that genotype A resistance to fluconazole was quite high as compared to other genotypes.^13^ The aim of this study was to determine the antifungal susceptibility and gene diversity of *C. albicans* isolated from a group of Jordanian women with VC.

## METHODS

Three hundred and twelve vaginal samples, positive for VC were collected from married women (18-55 years old) attending gynecology clinics in the following hospitals: Jordan, Specialty, and Islamic. Each of these hospitals contains more than 300 beds and out patient’s poly clinics. The study period lasted from July 2020 to December 2020. Demographic data and symptoms for each patient were derived from hospital files. High vaginal swabs were collected by gynecologists practicing in the respective hospital clinic.

### Ethical Approval:

A written consent was obtained from each woman and the work was approved by the Ethical Committee of the Islamic Hospital, Jordan. This approval was issued in September 12, 2020 under the number 4/2020/2493.

### Culture Procedure:

Sabouraud Dextrose Agar (SDA) medium was obtained from Oxoid- Irland and prepared by following the manufacturer’s instructions.^14^ After autoclaving, the medium was allowed to cool at 50°C before 5 mg/ dl of Chloramphenicol was added to it. The medium and the antibiotic were mixed and then poured into sterile petri dishes. Subsequently, the plates were stored at 2°C to 8°C for two weeks. Each collected vaginal swab was inoculated onto a plate of SDA in less than 24 hours of collection. All inoculated plates were incubated at 37°C for 48 hours and inspected for the presence of creamy to yellowish colonies which indicated the isolation of *Candida spp*.

### Chromogenic agar culture:

A single colony was picked up from the SDA plate and streaked on chromogenic agar (Biolife, Italy). The plate was then incubated for 24-48 hrs. at 37°C. Then, candida isolates were classified according to their color as proposed by the manufacturer of the medium. *Candida albicans* ATCC 90028 was used as a reference and gave good growth on the differential medium with greenish to blueish pigmentation.

### Sugar fermentation:

Various *Candida spp*. was identified by inoculating pure colonies taken from SDA into peptone liquid media (Oxoid- Irland). This medium contained phenol red as indicator, an inverted Durham’s tube and 2% of the following *Carbohydrates:* glucose, maltose, sucrose, Lactose, galactose, and trehalose; these sugars were incorporated separately into each peptone containing tube. Inoculated tubes were incubated at 37°C for 48 hours before the production of yellow color and gas were observed. Tubes that remained red were considered as negative for the respective sugar fermentation.

### Genomic DNA Extraction:

Genomic DNA extraction was achieved using i-genomic BYF DNA Extraction Mini Kit (South Korea). A colony of *C. albicans* was picked up from SDA plate and inoculated into a tube containing 5ml yeast peptone dextrose (YPD) broth (Condalab, Spain). The tube was incubated at 37°C for overnight to obtain an optical density of 600 nm. The detail of the procedure was typically the same as described in the manufacturer’s instructions manual. In brief, the first step was to break down the cell wall by lysis and to extract its intracellular contents. Once this content was obtained, it was treated with enzymatic solution to clear the DNA captured. The captured content was stirred with 80% ethanol to separate out any precipitate. The DNA was then collected and further immersed in a slightly alkaline buffer, “ready to use”. The quality and concentration of the DNA material in the sample was assayed spectrophotometrically.

### PCR Amplification:

Aliquots of 1 μl of the Genomic DNA Extract obtained for each *C. albicans* “purified as described above” was amplified by PCR on the bases of 25S rDNA using primers obtained from IDT (United States). For typing of *C. albicans* on the basis of ALT repeats two further primers were used. The sequence of the forward and reverse primers was described in Table-I. The program of the PCR was fixed as follows: one cycle of initial denaturation for 3 min, followed by 35 amplification cycles of denaturation at 94°C for 30 seconds, annealing at 59°C for 30 sec. and extension at 72°C for 30 seconds. The final extension was performed at 72°C for 10 minutes. The amplicon was separated by 1.5 % agarose gel electrophoresis supplemented with red safe. The system was run for 45 minutes at 450 voltage and then visualized using a gel documentation system (Cleaver Scientific, United Kingdom).

**Table-I:** List of PCR Primers used in this study.

Primer (P-1) 25S rDNA	Nucleotides sequence
CA-INT-L CA-INT-R	Forward: ATAAGGGAAGTCGGCAAAATAGATCCGTAA Reverse: CCTTGGCTGTGGTTTCGCTAGATAGTAGAT
Primer (P-11) ALT repeat	Nucleotides sequence
ASDcF Pcscr	Forward: TGATGAACCACATGTGCTACAAAG Revers: CGCCTCTATTGGTCGAGCAGTAGTC

### Consent to participate:

None of the data reported could lead to identification of patient.

### Antifungal susceptibility:

Yeast colonies were suspended in 0.85% sterile saline solution to adjust to 0.5 McFarland standard. This inoculum contained 1 × 10^6^ to 5 × 10^6^ cells per ml. A sterile cotton swab was used to spread evenly 500 μl fungal suspension on a 150-mm Petri dish containing Mueller-Hinton agar supplemented with 2% glucose and 0.5 μg/ml methylene blue dye. E-test strips “obtained from AB. Biodisk, Solna, Sweden” were placed on plates that had been dried for 15 minutes at room temperature. The strip end with a lower concentration of the antifungal was positioned first and placed in almost equal angles to the adjacent strip. The strips were purchased from AB. Biodisk, Solna, Sweden. After incubation for 48 hours, the test was read using the zone of inhibition to mark the point at which the ellipse-shaped growth intersects with the strip, indicating an MIC value.

Isolates with MICs ≤ 8 ug ml^-1^ for fluconazole (FLU) and 5 flucytosine (FC), ≤ 0.125 lg ml^-1^ for itraconazole (ITR) and ketoconazole (KET), and ≤1 lg ml^-1^ for Amphotericin B (AmB) were considered susceptible. Isolates with minimum inhibitory concentration (MICs) ≥ 64 ug ml^-1^ for FLU, ≥32 ug ml^-1^ for 5FC, ≥1 ug ml^-1^ for itraconazole (ITR) or KET, and ≥2 ug ml^-1^ for AmB were considered resistant.^12^ *Candida albicans* ATCC 90028 was used as a Control each time a susceptibility test was performed. The Validity of the Test was ascertained by comparing the susceptibility results with CLSI standards.^15^

### Statistical analysis:

One-way ANOVA and Chi-square tests were performed using the statistical SPSS package. Differences were considered significant at *P* < 0.05.

## RESULTS

A total of 312 positive vaginal swab samples for *Candida spp*. were included in this investigation. Positive samples were found to be more prevalent in the age group stratified between 25 and 31 years of age (Table-II). No statistical significance was found to exist between the occurrence of the infection and the various age groups. Growth pattern of *Candida spp*. isolated on chromogenic agar and their biochemical identification testing revealed the presence of four *Candida spp*. among the isolates (Table-III). The Table demonstrates that out of 312 *Candida spp*. tested, 175 (56.0%) were positive for *Candida albicans* while the remaining 137 (44%) harbored *Candida spp*. other than albicans.

**Table-II:** Number and percentage occurrence of VC stratified in various age groups.

Age group / years	Number of cases	Percent of cases
18-24	75	24
25-31	97	31
32- 38	50	16
39- 45	45	14.5
> 46	45	14.5

**Table-III:** Distribution and characteristics of isolated *Candida spp.* on Chromogenic agar.

Candida Species	Number of Isolates	Percentage (%)	Color on Chromo agar
*C. albicans*	175	56	Light green
*C. krusei*	105	33.6	Rose pink
*C. glabrata*	30	9.60	Pinkish purple
*C. tropicalis*	2	0.80	Metallic blue

Women included in this work complained of the following symptoms “in a descending order”:

Burning sensation during urination (46.4%), Pruritus (44.8%), vaginal erythema (24.8%), and dyspareunia (7.2%). The most astonishing observation was the high prevalence rate of VC in non-pregnant (88.8%) as compared to pregnant women.

**Fig.1:**
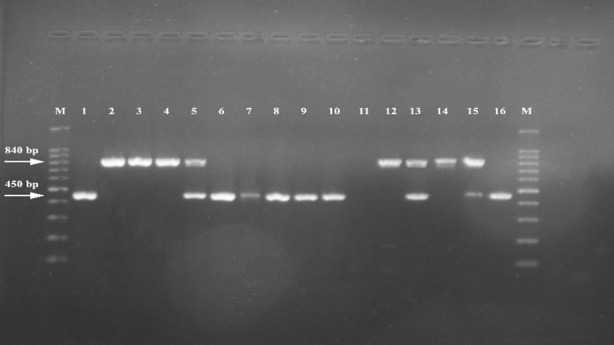
Amplification profiles of PCR products (25S rDNA) of Candida albicans isolates. (Genotype A: 450; genotype B: 840 bp; and genotype C: 450 and 840 bp) bp.

Using the CHI-SQUARE test it was calculated that there were no statistically significant results between VC and the detected symptoms except in case of pruritis and burning. In the current study, a total of 175 isolates were identified by phenotypic testing as *C. albicans*. These isolates were further typed using PCR with P-I and P-II primers. These primers were capable of determining the genotypes of the isolates based on variation in the 25S rDNA and repeated numbers of the ALT sequence (Table-IV and Table-V). The PCR amplified products of *C. albicans* using P-I, defined the DNA into three bands. These bands indicated the recovery of type A, B, and C of *Candida albicans*. Fig.1 shows that none of the PCR products in the current study demonstrated a band formation at the 1040 bp that corresponds to genotype D or at 1080 bp which indicated genotype E. However, band formation was obtained separately at 450 and 840 bp, in addition to the combined occurrence of two bands at 450 plus 840 bp; these bands indicated the recovery of A, B and C genotypes, respectively.

**Table-IV:** PCR expected results upon using P- I primer in relation to band size of the 25S rDNA.

Primer P-I	Band size (bp)	25S rDNA type
CA-INT-L and CA-INT-R	450	A
	840	B
	450, and 840	C
	1040	D
	1080	E

**Table-V:** PCR expected results upon using P-2 primers of ALT repeat

Primer P-II	Band size (bp)	ALT repeat
ASDcF Pcscr	526	1
	698	2
	870	3
	1042	4
	1214	5
	1386	6

**Fig.2:**
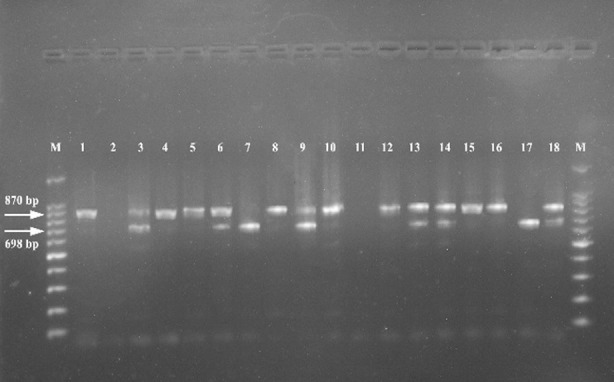
Genotyping identification for C. albicans strains by PCR targeting ALT repeats at the RPS using P-II primer.

When results obtained using the two genetic markers were combined together; the outcome was the identification of nine distinct genotypes (Histogram 1). On the basis of the repeated numbers of ALT sequence in the PCR amplification, the RPS profiles generated by primer P-II, indicated the detection of 4 subtypes of isolates (Fig.2). These were: Subtype 2 as 698 bp long (7.14% of isolates), subtype 3 as 870 bp long (45.70% of isolates), subtype 2/3 as “698 and 870” bp long (40.0% of isolates) and subtype 3/4 as “870 and 1040” bp, (7.14% of isolates).

**Histogram-1:**
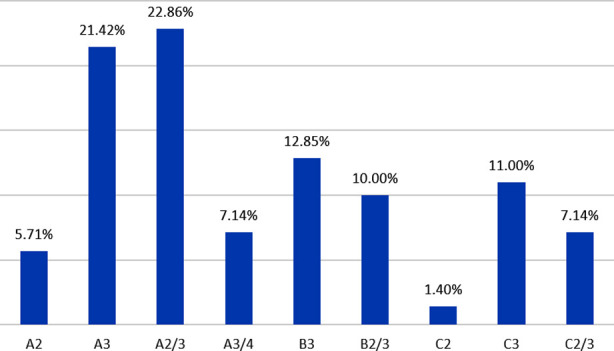
Percentage genotypic distribution of all C. albicans strain based on the two markers (P-I and P-II).

Antifungal susceptibility testing of the various genotypes showed variation in the susceptibility pattern of the different genotypes recovered. All isolates of genotype A, B and C were sensitive to flucytosine and fluconazole. However, only five out 40 genotype B isolates and 21 out of 35 genotype C isolates were sensitive to itraconazole. This antifungal was found to be the least effective inhibitory agent among the 5-antifungal studied (Table-VI).

**Table-VI:** Antifungal susceptibility and genotypes of Candida albicans strains from patients with vulvovaginal candidiasis.

Genotypes	Number	Amphotracin B	Itraconazole	Ketoconazol	Flucytosine	Fluconazole
A	100	98	95	100	100	100
B	40	40	5	38	40	40
C	35	35	21	35	35	35

## DISCUSSION

Only four *Candida spp*. were identified in this investigation as etiologic agent of vaginal candidiasis (Table-III); they were *C. albicans, C. krusei, C. glabrata*, and *C. tropicalis*. It was revealed that the highest frequency of vaginal candidiasis in the group of women studied was caused by *C. albicans* (56.0%). This observation is in agreement with most published literature from all over the world.^9^,^10^ In fact there is a variation in the type of Candida spp. recovered from vaginal swabs and this depends largely on the location as well as the population studied.

Predisposing factors for VC are numerous, including pregnancy and prior use of antibiotics. In the current work pregnancy was not found to be statistically significant factor in relation to the occurrence of infection, while the use of antibiotics was. High percentage (59%) of infected women diagnosed herein were found to have used antifungal drugs. This percentage is a lot higher than the 38% which was reported by Yano et al.^11^ In Jordan, antibiotics are officially considered as prescription drugs but in practice they are dealt with as over the counter drugs and this might explain why antibiotic use was found to be an important predisposing factor for vaginal candidiasis.

Among the women studied, the highest prevalence rate was detected in the age group stratified at 25 and 31 years of age. This is likely a reflection to the hormonal activity which takes place in this particular age that acts as a predisposing factor to infection. The relatively high proportions of VC in the postmenopausal (45 years or older) populations may reflect potential age-related to health conditions such as exogenous estrogen use to treat atrophy. Fischer and Bradford^12^ reported that nearly 50% of women above 50 years of age were diagnosed with VC attributed to hormonal replacement therapy.

Pruritus, burning, Erythema, and dyspareunia are among the most widely reported clinical features in VC. Burning, pruritis, and erythema were detected in (46%), (45%) and (25%) cases, respectively. The occurrence rate of these symptoms was a lot lower than those reported by Yano et al.^11^ but higher than those disclosed by Rathod et al.^13^ In the current work burning and pruritis were the only symptoms that gave statistically significant relationship with their number of occurrences.

For epidemiological reasons and treatment options, Odds et al.,^14^ suggested that it was of importance to clarify the taxonomic position of *C. albicans* and to determine the subpopulation within this species. *C. albicans* can be grouped into several genotypes by Southern hybridization, Pulsed-field gel electrophoresis (PFGE), and/ or Random Amplification of Polymorphic DNA (RAPD) techniques. From a cost point of view, Kanbe et al.^15^ suggested that identification of *C. albicans* by PCR, targeting the ALT repeat is more convenient than PFGE sequencing and RFLP techniques.

Tantivitayakul et al.^16^ indicated that *C. albicans* can be divided into four subtypes. This division is related to the presence and the size of transposable intron region in the large ribosomal subunit 25S rDNA. Based on this approach, these authors were able to identify different lengths of the PCR products as belonging to *C. albicans*, genotype A (450 bp), genotype B (840 bp), genotype C (450- and 840-bp), and genotype E (1400 bp). The same authors^16^ indicated the presence of association between *C. albicans* genotypes and fungal invasiveness as well as antifungal susceptibility.

To the best of our knowledge, reports on genotypic typing of *C. albicans* recovered from vaginal candidiasis in Jordan is seldom if at all presented. The current research contains information that has never been reported from Jordan. Genotype identification of *C. albicans* subtypes in this work were determined by PCR technique targeting 25S rDNA. The 175 *C. albicans* recovered herein were differentiated into three genotypes (A, B, and C). None of the isolates belonged to genotype (E or D) and genotype A accounted for 57.1%. These results contradicted those of Ali Shtayeh et al.^7^ and Gharaghani et al.^17^ who reported that the dominant *C. albicans* in their work belonged to Genotype C followed by Genotypes A and B. However, findings reported in our work regarding the genotypes are in agreement with those of Sawadogo et al.^18^

The ALT repeat sequence of the RPS revealed the presence of two patterns assigned to each of the three genotypes. In this study, nine types of *C. albicans* RPS were detected. These types were close to the 10 types identified by Sawadogo et al.^18^ but a bit higher than the 7 types reported by Amanloo et al.^19^ The Subtypes isolated in this study were A2 (5.71%), A3 (21.42%), A2/3 (22.86%), B3 (12.85%), B2/3 (10.0%), C2 (1.40%), C3 (11%) and C2/3 (7.14%). These findings are similar to those reported in other studies which characterized hundreds of *C. albicans* isolated from clinical samples including vaginal secretions, sputum, and blood 20. In the current investigation, A3/4 subtype was found to label a few isolates (7.1%). This observation was similar to the finding of Sawadogo et al.^18^ who detected this subtype in 6.3% of isolates. The dominance of subtype A3 among *C. albicans* isolated from clinical samples was also noted by Amanloo et al.^19^ and Iwata et al.^20^

Antifungal “in vitro” studies on the isolates revealed that vaginal candidiasis can be possibly treated to the same level of effectiveness using flucytosine and fluconazole. Whereas, Itraconazole was the least susceptible to all genotypes isolated. In fact, different results of susceptibilities were reported by different authors. For example, Zhu et al.^21^ found that genotype A strains were much less susceptible to flucytosine than either strains of genotype B or genotype C. On the other hand, Ali-Shtayeh et al.^7^ established that *C. albicans* genotype A were most resistant to fluconazole and flucytosine than B and C genotypes. Our results however are in agreement with those obtained by of Liu et al.^22^

### Limitations:

One of the limitations of this work was the lack of correlation between various genotypes of *C. albicans* with the established risk factors like uncontrolled diabetes and impaired immune response. It would have been of value to have performed some gene sequencing procedure to identify the genes responsible for antifungal resistance.

## CONCLUSION

This study showed that *C. albicans* was the highest in prevalence among *Candida spp*. recovered from cases of vaginal candidiasis in Jordanian women. Dominance of *C. albicans* was followed by *C. krusei*, *C. glabrata*, and *C. tropicalis*. Molecular genotyping characterized isolated *C. albicans* into three genotypes (A, B, and C). Using the ALT repeat sequences of the RPS, it was found that a genetic variation between *C. albicans* genotypes exist. Therefore, *C. albicans* depicted as etiologic agent of vaginal candidiasis were genetically diverse. Antifungal drugs were found to be *in vitro* effective against *C. albicans* to various levels depending on the genotype isolated. To our knowledge, this is the first report about the prevalence and antibiotics susceptibility of *C.albicans* isolated from vaginal candidiasis cases in Jordan

### Author`s Contribution:

**RMAG** Responsible and accountable for accuracy and integrity of the work. Helped in the design and supervision of the project. Analysis of result.

**RRMA** Design and carried out the experimental work, made the literature search. Helped in the interpretation and analysis of results.

**QMAS** Suggested and supervised the research project, prepared the original manuscript.

All authors discussed the results and revised the manuscript critically for important intellectual content.
